# Traumatismo Craneal por Maltrato: Nuestra Casuística Desde 2016 y Propuesta de Medidas de Prevención

**DOI:** 10.31083/RN38855

**Published:** 2025-03-07

**Authors:** Raquel Pérez Delgado, Jose Luis Peña Segura, María Zenaida Galve Pradel, Elena Javierre Miranda, Paula Almudena Madurga Revilla, Cintia Soro Lorente

**Affiliations:** ^1^Unidad de Neurometabolismo del Hospital Miguel Servet, 50009 Zaragoza, España; ^2^Unidad de Neonatología del Hospital Miguel Servet, 50009 Zaragoza, España; ^3^Pediatría de centro de salud de Atención Primaria Zuera, 50800 Zaragoza, España; ^4^Unidad de Cuidados Intensivos Pediátricos del Hospital Miguel Servet, 50009 Zaragoza, España

**Keywords:** lactante, encefalopatía aguda, traumatismo craneal, prevención, infant, acute encefalopathy, trauma, prevention

## Abstract

El traumatismo craneal por maltrato (“síndrome del lactante zarandeado”) se caracteriza por la triada encefalopatía aguda, hemorragias retinianas y sangrado cerebral, siendo la variante de maltrato infantil con mayor mortalidad y morbilidad neurológica asociada. Los cuidadores pueden no ser conscientes del peligro asociado con el zarandeo del lactante, por ello las medidas de prevención pueden jugar un papel muy importante. En nuestro centro observamos un aumento de casos entre finales de 2021 y primer trimestre del año 2022, hecho que condujo a diseñar un proyecto de calidad cuya finalidad sería hacer una campaña de prevención del síndrome del lactante zarandeado en nuestra comunidad autónoma diseñando un código QR con información que no sólo hace alusión a la prevención del lactante zarandeado sino también a otros consejos de crianza fomentando la parentalidad positiva.

## Abstract (en)

Abusive head trauma (AHT), commonly known as “shaken infant syndrome” is a severe form of child abuse characterized by the triad of acute encephalopathy, retinal hemorrhages, and cerebral hemorrhage, leading to high mortality and neurological complications. Many caregivers are unaware of the risks associated with shaking an infant, making prevention efforts essential. Our center observed an increase in AHT cases from late 2021 to early 2022, prompting the creation of a regional prevention campaign. This initiative introduced a scannable quick response (QR) code linking easily accessible educational materials on AHT prevention and parenting resources that promote safer caregiving practices.

## 1. Introducción

El traumatismo craneal por maltrato (Abusive head trauma) se refiere a todo aquel traumatismo que ocasiona lesiones intracraneales debido a un impacto directo infligido y/o zarandeo. Este término ha sustituido al anteriormente llamado “síndrome del lactante zarandeado”, que se caracteriza por la triada encefalopatía aguda, hemorragias retinianas y hematoma subdural [1]. Se trata de la variante de maltrato infantil con mayor mortalidad y morbilidad neurológica asociada [2].

La clínica de presentación en los servicios de urgencias pediátricos puede ser muy variada e inespecífica, con escasos o nulos signos externos traumáticos. Ninguna lesión es patognomónica, se requiere un alto grado de sospecha para hacer un diagnóstico. Debemos sospecharlo si hay un retraso injustificado en la consulta médica, se atribuye la clínica o lesión a un accidente como puede ser una caída, a veces atribuida a un hermano. El agresor en orden de frecuencia suele ser en primer lugar el padre o la nueva pareja de la madre, cualquier otro cuidador y, en último lugar, la madre. Se debe tener claro que un traumatismo craneal leve y la manipulación normal del niño no producen estas lesiones propias del zarandeo.

Los movimientos de aceleración y deceleración que se dan durante el zarandeo van a producir un desgarro de los vasos del espacio subdural y las hemorragias retinianas. Ello sumado a las características anatómicas del lactante menor de 1 año (poco tono cervical y mayor volumen de la cabeza) van a favorecer su vulnerabilidad para este tipo de lesiones. Los lactantes hipotónicos y con macrocefalia asociada a mayor espacio pericerebral van a tener más alto riesgo.

Los cuidadores pueden no ser conscientes del peligro asociado con el zarandeo del lactante, es por ello que las medidas de prevención pueden jugar un papel importante para evitar las secuelas y riesgo de mortalidad asociados a estos casos.

En nuestro centro observamos un aumento de casos entre finales de 2021 y primer trimestre del año 2022, hecho que condujo a diseñar un proyecto de calidad cuya finalidad sería hacer una campaña de prevención del síndrome del lactante zarandeado en nuestra comunidad autónoma.

## 2. Material y Métodos

Se realizó una búsqueda de casos de “síndrome de lactante zarandeado” en la base de datos de la Unidad de Neuropediatría de nuestro centro, cuyo registro de pacientes se inició en el año 1990, con un periodo de búsqueda desde comienzos del año 2016 hasta la actualidad.

Se han tenido en cuenta edad de presentación, sintomatología al debut y gravedad (necesidad o no de ingreso en UCI), pruebas complementarias realizadas, morbilidad, tratamientos y secuelas a largo plazo.

## 3. Resultados

Obtuvimos 7 casos en el periodo de estudio, estando los últimos 4 concentrados entre noviembre de 2021 y abril del 2022.

Los casos se presentan en la Tabla 1.

**Tabla 1.** **Resultados, casos codificados como síndrome de lactante zarandeado en nuestra base de datos**.

	Edad	Ingreso UCI	Clínica	Pruebas	Secuelas	Evolución	TRatamiento
Caso 1	2 m		Hipoactiva	TC craneal	Normalidad		
2016			Aumento PC	FO			
			Vómitos	Serie ósea			
Caso 2	7 m	Si	EA	TC craneal	Normalidad		
2017				FO			
				Serie ósea			
Caso 3	4 m	Si	EA crisis	TC craneal	Epilepsia	Otra CCAA	Valproato
2018				FO	Ceguera		
				Serie ósea	Tetraparesia		
Caso 4	5 m		Aumento PC	TC craneal	Normalidad	Exitus inesperado	
2021				FO			
				Serie ósea			
Caso 5	3 m	Si	EA crisis	TC craneal	Epilepsia	West	Levetiracetam
2022				FO	Tetraparesia		Vigabatrina
				Serie ósea			ACTH
Caso 6	4 m	Si	EA	TC craneal	Ceguera		
2022			Hematomas	FO			
				Serie ósea			
Caso 7	2 m	Si	EA crisis	TC craneal	Grave encefalopatía	Exitus	
2022				FO	difusa		
				Serie ósea			

EA, Encefalopatía aguda; PC, Perímetro cefálico; FO, Fondo de ojo; UCI, Unidad de Cuidados Intensivos; TC, CCAA, Comunidad Autónoma; ACTH, Hormona adrenocorticotropa.

La edad de los pacientes fue de dos a siete meses, siendo la clínica predominante la encefalopatía aguda acompañada de crisis convulsivas. El único caso que no presentó encefalopatía aguda (caso 4) fue remitido por su pediatra para estudio por aumento de perímetro cefálico detectado en una revisión rutinaria, ya que la última medida se salía del máximo percentil de normalidad en más de dos desviaciones estándar y la curva de crecimiento cefálico era anormalmente ascendente. Cinco de 7 casos (71%) fueron ingresados a su llegada en UCI-Pediátrica.

En todos los casos se hizo TC craneal y fondo de ojo en el momento agudo, así como serie ósea esquelética en diferido. Se realizó parte al juzgado y consulta a trabajo social también en el 100% de los casos. Todos tuvieron hemorragias retinianas y salvo el caso 4 todos tuvieron sangrado intracraneal (en el caso 4 los hallazgos consistieron en un gran aumento del espacio pericerebral y dudosos restos de sangrado en el espesor del Líquido cefalorraquídeo (LCR)).

Sólo en un caso el agresor reconoció el zarandeo (el padre); se trató del único caso que presentaba lesiones externas visibles, hematomas faciales.

Dos niños fueron éxitus, uno de ellos durante los primeros días del ingreso como consecuencia de las importantes secuelas y el caso 4 estando ingresado para estudio sufrió una parada cardio- respiratoria en circunstancias no aclaradas (se sospechó asfixia por parte del cuidador que no pudo ser demostrada).

Dos pacientes tuvieron epilepsia sintomática a las lesiones, una de ellas espasmos infantiles epilépticos. Otro paciente no tiene epilepsia ni secuelas motoras, pero quedó con importante disfunción visual.

## 4. Discusión

La edad media de la edad de presentación del síndrome de lactante zarandeado suele ser entre los 2 y los 6 meses, época que coincide con el pico de mayor incidencia de llanto inconsolable, principal factor desencadenante de este tipo de maltrato.

Es frecuente el desconocimiento por parte de padres y cuidadores de que sacudir o zarandear a un bebé puede ser tan peligroso, siendo en muchas ocasiones una acción no premeditada y en momentos de desesperación ante la imposibilidad de calmar el llanto del niño.

En E.E.U.U. y otros países europeos las campañas de prevención son muchas desde hace años (carteles informativos, anuncios en las marquesinas de los autobuses..) y existen muchos artículos publicados en revistas médicas que hablan de prevención [3, 4, 5, 6].

En España se ha tenido la impresión de un aumento de casos a nivel general coincidiendo con la pandemia por Covid19 y en varios hospitales se han diseñado estrategias de prevención, tratándose en la mayoría de los casos de folletos informativos ó vídeos.

En nuestro centro como parte de un proyecto de mejora de calidad se ha diseñado un código QR que no sólo hace alusión a la prevención del lactante zarandeado sino también a otros consejos de crianza y se fomenta la parentalidad positiva. Se ha preferido este método por ser visualmente más atractivo, en la línea de la tecnología actual, que además supone un ahorro en papel. Se han colgado carteles en las habitaciones de la maternidad y se ha propuesto que desde los centros de salud se chequee el conocimiento por parte de los padres de estas medidas.

## 5. Conclusiones

Debemos sospechar maltrato o zarandeo ante todo lactante que se presenta con encefalopatía aguda a los servicios de urgencias pediátricos.

La sintomatología en la mayor parte de los casos va a ser grave y serán niños candidatos a ingreso en unidades de cuidados intensivos.

Este tipo de maltrato cursa con alto riesgo de mortalidad y secuelas neurológicas graves y se puede presentar de forma recurrente.

Los cuidadores pueden ser desconocedores de los riesgos y por ello son importantes las medidas de prevención primaria.

Es muy importante la colaboración entre los diferentes profesionales que intervienen en la asistencia de los recién nacidos y lactantes, y todos debemos ir en la misma línea evitando mensajes contradictorios y reforzando unos conocimientos básicos de crianza y cuidados básicos que aseguren la seguridad de nuestros niños.

## Data Availability

Los datos clínicos y la neuroimagen de los pacientes se encuentran disponibles en la base de datos de la unidad de Neurometabolismo del hospital Miguel Servet de ZAragoza.

## Contribuciones de los Autores

RPD, diseñó el estudio y realizó la investigación, analizó los datos; JLPS, diseñó el proyecto de calidad ”prevención del síndrome del lactante zarandeado”; MZGP, participó en el proyecto de prevención representando a la Neonatología y Maternidad, plantas donde se difunde el código QR con la información para padres; EJM, participó en el proyecto de prevención representando a la Pediatría de Atención Primaria, ya que en los centros de salud se chequea la adecuada información de los padres y cuidadores; PAMR, participó en el proyecto de prevención representado a la Unidad de Cuidados Intensivos, donde ingresan la mayor parte de estos pacientes en situación de encefalopatía aguda; CSL, participó en el proyecto de prevención representando a la Neonatología y Maternidad, plantas donde se difunde el código QR con la información para padres. Todos los autores contribuyeron a los cambios editoriales en el manuscrito. Todos los autores leyeron y aprobaron el manuscrito final. Todos los autores han participado de manera suficiente en el trabajo y han aceptado ser responsables de todos los aspectos del mismo.

## Aprobación Ética y Consentimiento Informado

No aplicable.

## Agradecimientos

No aplicable.

## Financiación

Esta investigación no recibió financiación externa.

## References

[1] Lafuente-Hidalgo M, Ranz-Angulo R, Martín-Cuartero J, Navarra Vicente B, López Pisón J (2017). Síndrome del niño zarandeado. Traumatismo craneal no accidental. *Form Act Pediatr Aten Prim*.

[2] Lai CD, Marret MJ, Jayanath S, Azanan MS (2023). Abusive head trauma in infants: An observational single centre study comparing developmental and functional outcome between 18 months and 5 years. *Child Abuse & Neglect*.

[3] Fortin V, Ortiz ARDA, Marq AD, Mostermans E, Marichal M, Bailhache M (2024). Childminder knowledge of shaken baby syndrome and the role played by childminders in prevention: An observational study in France. *Archives De Pediatrie: Organe Officiel De La Societe Francaise De Pediatrie*.

[4] Chang HY, Chang YC, Chang YT, Chen YW, Wu PY, Feng JY (2024). The Effectiveness of Parenting Programs in Preventing Abusive Head Trauma: A Systematic Review and Meta-Analysis. *Trauma, Violence & Abuse*.

[5] Laurent-Vannier A (2022). Shaken Baby Syndrome (SBS) or Pediatric Abusive Head Trauma from Shaking: Guidelines for Interventions During the Perinatal Period from the French National College Of Midwives. *Journal of Midwifery & Women’s Health*.

[6] Dean JR, Kaczor K, Lorenz D, Mason M, Simonton K (2024). Characteristics of child abuse fatalities: Insights from a statewide violent death reporting system. *Child Abuse & Neglect*.

